# Optimization and enhancement of H&E stained microscopical images by applying bilinear interpolation method on lab color mode

**DOI:** 10.1186/1742-4682-11-9

**Published:** 2014-02-06

**Authors:** Kaya Kuru

**Affiliations:** 1 IT Department, Gulhane Military Medical Academy, Etlik, Ankara, Turkey

**Keywords:** H&E staining technique, Bilinear interpolation, LAB color, Microscopic digital images, Koehler illumination, Non-uniform illumination

## Abstract

**Background:**

Hematoxylin & Eosin (H&E) is a widely employed technique in pathology and histology to distinguish nuclei and cytoplasm in tissues by staining them in different colors. This procedure helps to ease the diagnosis by enhancing contrast through digital microscopes. However, microscopic digital images obtained from this technique usually suffer from uneven lighting, i.e. poor Koehler illumination. Several off-the-shelf methods particularly established to correct this problem along with some popular general commercial tools have been examined to find out a robust solution.

**Methods:**

First, the characteristics of uneven lighting in pathological images obtained from the H&E technique are revealed, and then how the quality of these images can be improved by employing bilinear interpolation based approach applied on the channels of Lab color mode is explored without losing any essential detail, especially for the color information of nuclei (hematoxylin stained sections). Second, an approach to enhance the nuclei details that are a fundamental part of diagnosis and crucially needed by the pathologists who work with digital images is demonstrated.

**Results:**

Merits of the proposed methodology are substantiated on sample microscopic images. The results show that the proposed methodology not only remedies the deficiencies of H&E microscopical images, but also enhances delicate details.

**Conclusions:**

Non-uniform illumination problems in H&E microscopical images can be corrected without compromising crucial details that are essential for revealing the features of tissue samples.

## Introduction

Pathology is the study of cells and tissues in which structural and functional changes take place. Pathologists examine tissue slices,^a^ cytology, body fluids for abnormal levels of chemicals and the presence of crystals; they carry out molecular studies to diagnose diseases under the microscope. Identifying abnormal cells and the morphology of tissues under the light microscope are essentially conducted by histological staining techniques. Among these, Hematoxylin & Eosin (H&E) technique is one of the most common employed techniques [1]. This technique has become the oversight method of first choice for most practitioners of normal histology and histopathology since first introduced in 1876 [2]. The technique allows both enhancing contrast and discerning between nuclei and cytoplasm in tissues by staining them in different colors. These colors are namely blue by applying hemalum, which is a complex formed from aluminium ions and oxidized hematoxylin, and reddish (namely red, pink and orange)by applying an aqueous or alcoholic solution of eosin. This bi-coloring process helps to better depict the microscopic morphology of tissues and cells, and eases the diagnosis through the microscope even though dyes are highly inconsistent for various reasons^b^[2].

With the recent advances in digital imaging, the latest digital cameras coupled with powerful computer methods offer not only better image quality that is comparable with traditional silver halide film photography, but also greater flexibility for image manipulation and storage; hence, they are increasingly being used for image capture for microscopy – an area that demands high resolution, color fidelity and careful management [3]. Acquiring better images as well as depicting slices better through microscopes depends essentially on three interrelated components: one of which is the proper alignment of the filament and condenser; the other one is the quality of microscopes; and the last one is the regular maintenance of microscopes including calibration. These issues are explained briefly in the following paragraph.

One of the most misunderstood and generally neglected concepts in optical microscopy is the proper configuration of the microscope with regards to illumination, which is a critical parameter that must be fulfilled in order to achieve optimum performance [4]. The intensity and wavelength spectrum of light emitted by the illumination source is of significant importance, but even more essential is that light emitted from various locations on the lamp filament be collected and focused at the plane of the condenser aperture diaphragm [4]. In this respect, the proper alignment of the filament and condenser is an essential requirement to establish good Koehler illumination. Moreover, when working with living cells it is imperative to avoid relatively high light intensities and long exposure times that are typically employed in recording images of fixed cells and tissues (where photo bleaching is the major consideration) [5]. The misalignment of the filament and condenser along with low light situations lead to serious illumination problems; this reduces the quality of the digital images, in particular those obtained from the H&E technique.^c^ Moreover, it should be noted that there are several types of microscopes ranging from a good quality that is unbelievably expensive to a less quality that is much less expensive. Quality of calibration is very much dependent on the quality of microscopes. Some less quality microscopes may not produce satisfactory results even though they are calibrated well which leads to an illumination problem at any circumstances. Since bulbs used in microscopy as an illuminator have a limited life, they should be changed regularly in terms of the hours they are used. Therefore, regular maintenance of microscopes is a vital issue both for depicting slices better and for acquiring better images. In this empirical study, an effective approach is presented by addressing this specific problem that is commonly encountered by pathologists and histologists.

The methodology presented in this study not only addresses the deficiencies of the general off-the-shelf commercial methods in terms of enhancing H&E images, but also outperforms better than the specific methods that have been established for remedying the particular illumination problem in microscopic images.

## Methodology and results

### Background

**Figure 1:**
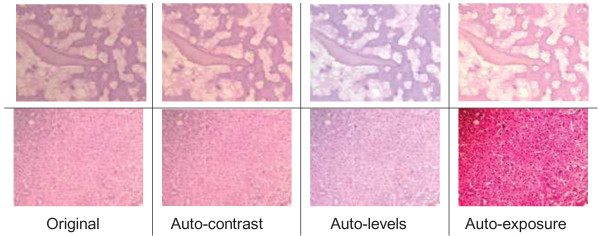
**Correction of illumination using different methods.** Two sample H&E images before and after employing some common optimization methods in RGB color model. These popular methods are auto-contrast, auto-levels and auto-exposure.

In order to remedy defects on digital H&E images, pathologists rely on various tedious and time-consuming image processing methods that work in RGB color model to optimize their images. The RGB color model is an additive color model in which red, green, and blue light is added together in various ways to reproduce other colors; it is device dependent, meaning that the output may vary from one device to another. Optimizing images is usually time consuming and mostly ad-hoc process; sometimes even experienced pathologists may not be able to get images in correct appearance even through great effort. For instance, Figure 1 shows two sample pathological images both in their original form and in their processed form using three different prominent optimization procedures that are embedded in most of the popular image processing tool. These applied procedures are auto-contrast, auto-levels^d^ and auto-exposure respectively. Auto-contrast builds a histogram of the image and spreads out the values so the whole tonal-range is being used; auto-levels adjusts brightness and contrast to produce a balanced image with a good range of color intensities; and auto-exposure measures the darkest and lightest points in the image and adjusts the brightness accordingly. Note that, the effect of auto-contrast is minimal, and a color shift is apparent in the other optimized images. The appearance of the image regarding the color shift not only disturbs the appearance, but also results in considerable information loss for hematoxylin and eosin stained sections which can be perceived even by naked eye. Moreover, these ad-hoc methods are not successful to correct not-balanced poor Koehler illumination spreading through the image differently which is explained in the following section with several examples in detail. This unbalanced (non-uniform) spread of illumination problem is of great importance as a commonly seen problem not only in ordinary microscopic images, but also in images used in pathology textbooks and publications.

**Figure 2:**
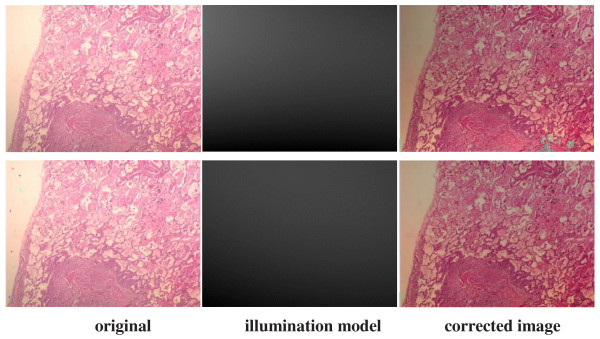
**Correction of the H&E image using “*shading correction*” plug-in in ImageJ.** First row depicts the outcomes acquired using the automatic correction utility of the method: background points are selected by the utility. The images at the second row depict the semi-automatic correction utility: background points are selected by the user as selected point can be seen on the image. Some artifacts in blue sections are evident in both corrected images.

**Figure 3:**
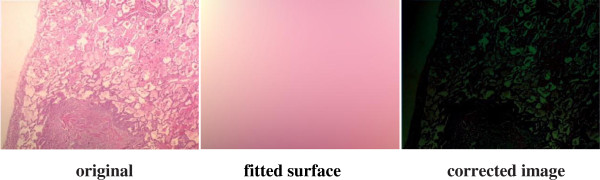
**Correction of the H&E image using “*polynomial_fit*” plug-in in ImageJ.** The fitted surface obtained by the method is subtracted from the original image using “Image Calculator” method in ImageJ to generate the corrected image. The result is not satisfactory at all.

**Figure 4:**
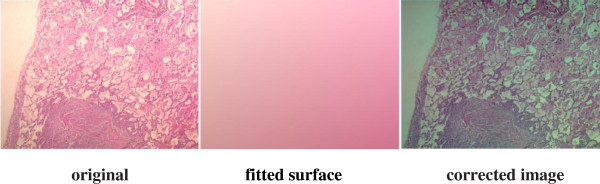
**Correction of the H&E image using “*fit polynomial*” plug-in in ImageJ.** The fitted surface is subtracted from the original image using “Image Calculator” method in ImageJ to generate the corrected image. The non-uniform illumination problem is transformed into a uniform illumination problem.

**Figure 5:**
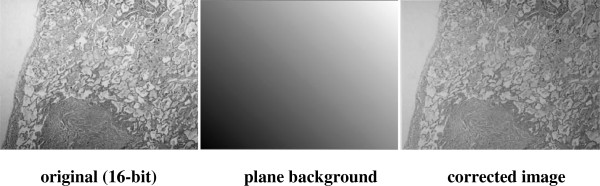
**Correction of the H&E image using “*nonuniform background removal*” plug-in in ImageJ.** This plug-in only works with 16-bit images, thus, original image is converted into 16-bit image and non-uniform correction is performed on this 16-bit original image. The uneven lighting on the image is corrected considerably.

**Figure 6:**
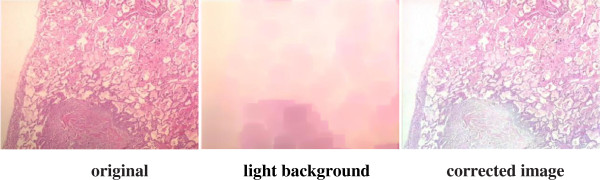
**Correction of the H&E image using “*subtract background*” method as a process in ImageJ tool.** Densely stained sections (i.e. lower middle) are accepted as a background noise and these sections in the generated corrected image get much brighter than expected, thus, many delicate details are lost in terms of the color shift in the image.

To overcome these problems, two main approaches specific to staining techniques have been proposed so far, one of which is the priori correction and the other one is posteriori correction. Various digital image analysis methods have been described [6-11] regarding these two approaches, and some of these methods have been plugged in several software tools such as Matlab, Fiji ImageJ and ImmunoRatio [12]. Some priori measurement must be carried out each time with the priori correction approach: the settings of the camera and the microscope light should be adjusted at the beginning and measured background noise should be subtracted from each image to detect better images. In this respect, the priori correction techniques require time consuming and tedious procedures. For instance, for priori color correction with Fiji ImageJ firstly, a shot is captured after blocking the light tube to acquire hot pixels on white/gray glass background; another shot for background is captured without blocking the light tube to compensate background illumination for once. Secondly, a shot is captured for each specimen to apply correction: hot pixels are subtracted from both the background image and the image captured for specimen by using the utility named “*Image Calculator*”; lastly, the subtracted image for specimen is divided by the subtracted background image using the utility named “Calculator Plus” with some parameters to generate the corrected image. For priori color correction with ImmunoRatio using “*Camera Adjustment Wizard*”, firstly, a shot is captured on white/gray glass background to detect the background noise; another shot for the specimen stained only by blue (hematoxylin) is captured. Secondly, background noise is removed from the image captured for the specimen stained by hematoxylin to acquire a better image. ImmunoRatio works for several staining protocols [12], but not for H&E staining protocol. The background noise changes both with the heated environment caused by the microscope light as the time goes by and with the adjustment of magnification parameters during examination under the microscope, thus, an ideal image is still difficult to acquire using priori techniques. On the other hand, for the posterior correction, some methods (one of which is proposed in this manuscript) such as “*shading correction*”, “*polynomial_fit*”, “*fit_polynomial*”, “*nonuniform background removal*” and “*subtract background*” are embedded in various software tools to eliminate time consuming and tedious work steps in priori techniques. Our sample images are examined employing these methods to observe their effects on H&E images. For “*shading correction*” method as a plug-in embedded in ImageJ,^e^ several points are selected either by the user or by the method automatically to acquire the uneven illumination problem (background noise) and the background noise is removed from the image in terms of the polynomial correction. The results of “shading correction” method regarding the automatic and semiautomatic procedures on one of our example images are depicted in Figure 2. Some artifacts in blue sections are evident in both corrected images and the non-uniform illumination problem is not corrected using this technique. For “*polynomial_fit*” method as a plug-in embedded in ImageJ,^f^ the orthogonality relation of the Legendre polynomials is used to expand an image as a double sum of those functions. The sum is then evaluated to produce an image that approximates a projection onto the space of polynomial images. The results of “*polynomial_fit*” are depicted in Figure 3. The results of the method is not satisfactory at all for H&E images. For “fit_polynomial” method as a plug-in embedded in ImageJ,^g^ “*shift values to display range*” uses the mean value of the current display range, (max + min)/2, as zero for the subtracted output image. The results of “*fit_polynomial*” are depicted in Figure 4. The non-uniform illumination problem is transformed to a uniform illumination problem with this method. For “*nonuniform background removal*” method as a plug-in embedded in ImageJ, a least-square fit of background samples within the image to one of two intensity profiles: 1) a plane, or 2) a 2D cubic polynomial is found and the estimated background is subtracted from the input image. One of the drawbacks of this method is that it only works with 16-bit images even though non-uniform illumination problem is corrected considerably. The results of “*nonuniform background removal*” are depicted in Figure 5. Finally, for “*subtract background*” method [6] embedded in the ImageJ tool, an example is displayed in Figure 6. In the example, densely stained sections are accepted as a background noise and these sections in the generated corrected image get much brighter than expected, thus, many delicate details are lost in terms of the color shift in the image. This method introduces some image artifacts as well.

**Figure 7:**
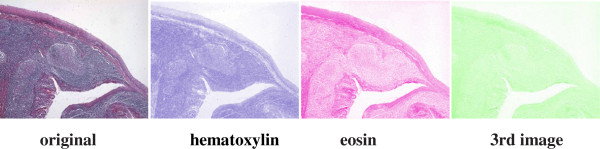
**Color deconvolution of a H&E image using ImageJ tool.** The vectors specified for red, green and blue layers for the color deconvolution do not perfectly match the stains in this image since 3^rd^ image is not all white.

On the other hand there is a method, named “color deconvolution” (CD) to decompose the colors of stained images. CD that works in RGB color mode was introduced by Ruifrok and Johnston [1]. This method is embedded in various image processing tools such as ImmunoRatio and ImageJ to realize several objectives. ImmunoRatio tool has inherited the function of CD and calculates the percentage of stained areas using cyan-magenta-yellow-black (CMYK) color model, hue-saturation-intensity color model and CIE 1976 L*u*v (CIELUV) color model whereas CD in Fiji ImageJ introduces three RGB images by separating the staining components into two or more overlapping stains using RGB color model [12]. Each of these components is constructed by adding red, green and blue channels at different levels. Several color correction methods can be applied on these deconvolution images. However, it is important to note that the reliability of measurement depends mainly on the quality of the sample input, the use of standard methods, inclusion of standard samples and prevention of signal saturation [1]. Moreover, applying CD should be adapted to various staining protocols, microscope setups, digital camera models, and image acquisition settings [12]. Therefore, the decomposed images depend on some assumptions since all of these settings cannot be incorporated into the CD algorithm. In this respect, Rabinovich [13] addresses the discrepancy between the decomposition and the ground truth images which is caused by the chemical interaction between the various dyes used for staining. For instance, the decomposed images of a specimen stained by H&E technique are depicted in Figure 7.^h^ In this example, the vectors specified for red, green and blue layers for the color deconvolution do not perfectly match the stains in this image since 3rd image is not all white; therefore, new vectors should be determined to achieve a more accurate stain separation, depending on the stains, settings and methods we use. However, new vectors can be determined by priori methods mentioned above requiring tedious and time consuming steps.

**Figure 8:**
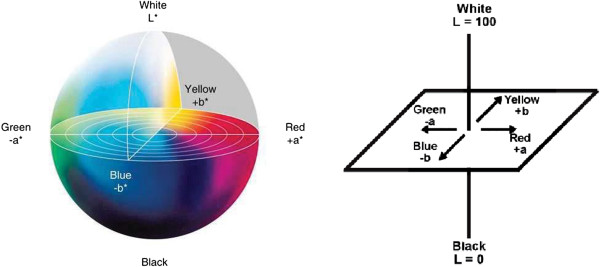
**The CIE Lab color dimensionality.** The dimensionality of CIE Lab color model is illustrated in two drawings.

**Figure 9:**
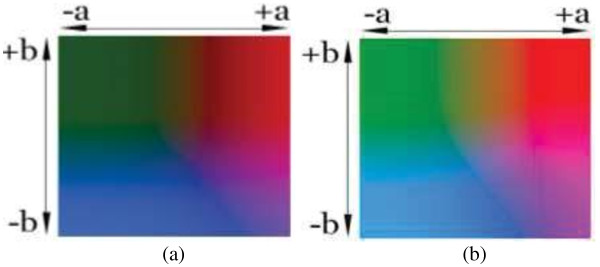
**Lightness in CIE Lab model.** The images show the colors in a* and b* dimensions with L* values of **(a)** 25 and **(b)** 50 respectively. All the pixel values in the L* channel of the left image **(a)** have a value of 25 while the pixel values have a value of 50 for the right image **(b)**.

All retrospective methods developed so far make assumptions about the image characteristics; thus, correcting stained images with a priori method has become advantageous to posteriori methods regarding the measurements in real environment, but it is very time consuming and tedious. Moreover, it seems impossible to employ a priori method on the images captured previously. The difficulty stems from the fact that the RGB color model does not allow modifying the illumination of an image without altering the information stored in the red and blue channels of the image. Since, nuclei of cells are colored in blue and cytoplasms are colored in red in the H&E staining technique, illumination corrections in RGB color model inherently affect the data that the pathologists are interested in. In particular, some delicate details become barely perceptible impair the elaborateness of the images as illustrated in Figures 1, 2, 3, 4, 5, and 6. In order to address these issues, a new methodology that is not restricted to fixed colors in H&E images is required to be established since colors of H&E staining technique are not static as specified in the section of introduction. This brings the need for a solution in which the lightness and the chromaticity of pixels in an image can be changed independent of each other in terms of the characteristics of H&E technique. One possible option, is to use the CIE 1976 (L*, a*, b*), or CIE LAB, color space. The CIE LAB color space aims to conform to human vision and can describe all the colors visible to the human eye. It is a device-independent model established by the International Commission on Illumination [14,15]. The coordinates of CIE LAB color model, L*, a* and b*, represent the lightness of the color, its position between red/magenta and green, and its position between yellow and blue respectively (Figure 8). L* values range from 0 to 100; a value of 0 yields black whereas a value of 100 yields diffuse white. Negative a* values denote green while positive values denote magenta; similarly, negative b* values indicate blue and positive values indicate yellow. L* component closely matches human perception of lightness. Thus, it is possible to make accurate color balance corrections by modifying output curves in a* and b* components, or/and by adjusting the lightness contrast using the L* component. As an example, the distribution of colors in a* and b* dimensions for two different L* values, 25 and 50, are shown in Figure 9. Despite the dramatic change in the lightness, the color information is stable. By taking advantage of this property and the characteristics of the uneven lighting in H&E images, a novel approach is proposed not only to correct the illumination problems in images but also to optimize them without losing any delicate detail which is essential for diagnosis.

To summarize, several off-the-shelf methods particularly established to correct illumination problem in H&E images along with some popular general commercial methods have been examined to find out a robust solution. Our evaluation of these previous studies has formed the basis for the software requirement analysis that consists of architectural, structural, behavioral and functional requirements. The methodology proposed in this study will be described in detail and its effectiveness will be demonstrated on sample microscopic images in the following two sections.

### Correction of illumination

**Figure 10:**
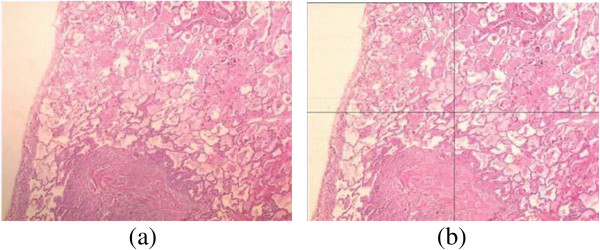
**Two images of the same specimen with different alignment of the filament and condenser. (a)** There is a non-uniform illumination problem for the first image as illustrated in Figure 12(a). L* values change throughout the image regarding the non-stained white areas; **(b)** The second image has some uniform illumination problem as illustrated in Figure 12(b), even though the alignment of the filament and condenser is better and the calibration of the microscope that is a good quality has been performed recently. The L* values in four quadrants of the image (as it is divided in four equal parts) regarding the non-stained white areas are almost equal to each other, but, smaller than 100 that is the desired value for the non-stained sections.

**Figure 11:**
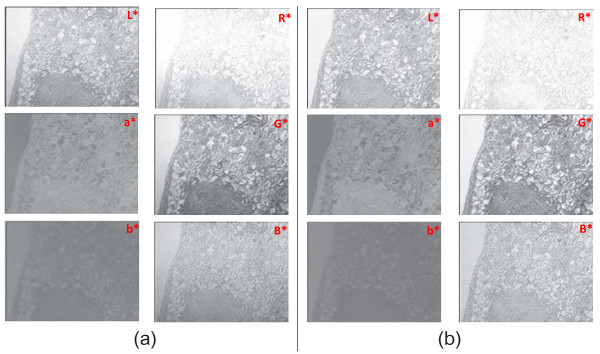
**Channels of the Lab and RGB color models. (a)** L*, a* and b* (first column, from top to bottom), and red, green and blue (second column, from top to bottom) components of the image in Figure 10(a); **(b)** Likewise, L*, a* and b* (first column), and red, green and blue (second column) components of the image in Figure 10(b). The components are specified at the upper right of the images as well.

As mentioned in the previous section, the CIE LAB color model provides a suitable and convenient way to represent and manipulate H&E images. Lab color lets you split the image into completely independent layers for brightness and color information (described as two independent chromaticity layers a* and b*). Each of these layers as representing the color information of H&E (nuclei and cytoplasm) sections can be processed individually and they can be combined back together into a modified color image by maintaining its own original colors in the channels whose colors are not processed. In order to remedy the illumination problems in these images, the remaining missing piece of the puzzle is the characteristics of uneven lighting that arises in such images. For this purpose, a large set of pathological images obtained by the H&E technique has been analyzed.^i^ Figure 10 shows two images of a sample specimen with different alignment of the filament and condenser. These images should have a white area on the left side; because, there is no cell stained by the H&E technique at that area. In other words, the areas not stained by H&E technique must be comprised of exact white pixels which are used as a gold standard in our study to detect the pattern of uneven lighting. In the first image, a gradient of increasing intensity (getting darker toward the bottom) which is caused by uneven lighting can be observed easily. The second image, on the other hand, has a relatively uniform illumination and does not suffer from such a problem; however, it still has an illumination problem spreading almost uniformly all over the image. The L*, a* and b*, and red, green, and blue components of both images in CIE LAB and RGB color models are presented in Figure 11. When these channels are examined individually, especially channels at the left side of Figure 11 in which an uneven lighting is evident, it can be perceived that the illumination problem is only carried out to the L* channel for Lab model where as this problem is shared in red, green and blue channels for RGB model (please notice the left side of the images to perceive the illumination problem). Therefore, we can easily conclude that correction of any lighting problem can be established by working on L* channel rather than a* and b* channels for Lab mode. On the other hand, a correction procedure is required for each channel for RGB mode to correct any illumination problem in images which unavoidably causes loss of some delicate crucial details in H&E sections of images. That’s why, Lab color mode is chosen in this study for the correction of uneven lighting in H&E stained microscopical images.

**Figure 12:**
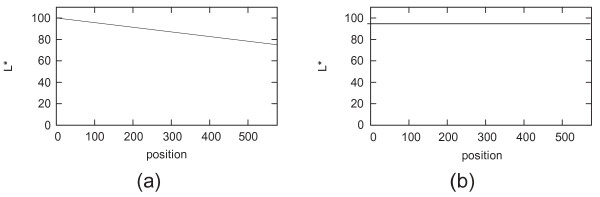
Distribution of L* values in H&E images. The distribution of L* values of the pixels in the left-most area of the images in Figure 10 is presented. The lines in the figures indicate the L* pixel values from the lower left corner to the most left upper corner: **(a)** depicts the distribution of the image in Figure 10 (a); **(b)** depicts the distribution of the image in Figure 10(b).

When the lightness component, i.e. L*, of the images are examined more carefully in Figure 10, it is seen that the L* values of pixels on the left side of the first image decrease linearly along the edge of the image (Figure 12a); this trend spreads equally all over the image. As expected, the L* values of the pixels in the same region of the second image stay almost constant (Figure 12b). Many images in a large data set have been analyzed to detect the pattern of the uneven lighting. This analysis reveals that each pathological digital image with uneven lighting more or less has a similar linear pattern depending on the distinct positions of either the illumination apparatus or the microscope. From a global point of view, this common pattern tells us that the L* values of pixels in the CIE LAB color model and the relationship between them, for example, near the corners, indicate how the uneven lighting spreads through the image, either increasingly or decreasingly with a different angle with the XY plane. Given an image and a set of points on that image that are supposed to be white (i.e. the L* values should equal to 100), a mask that approximates the distribution of the uneven lighting can be generated. The image should be first converted to the CIE LAB color model, and then interpolating and exterpolating L* values of these points should be subtracted from a reference value, in most cases 100, to create the mask. The mask has a subtraction value for each pixel in the image. Secondly, this mask is subtracted from the L* channel of the image and the image is converted back to the RGB color model. This processes help reduce the uneven illumination significantly, in most cases eliminate it. The procedures for converting an image from the RGB color model to the CIE LAB color model and vice versa are provided in the appendix. Note that, during this process a* and b* channels of the image, which comprise information about nuclei and cytoplasm in tissues, are not altered.

**Figure 13:**
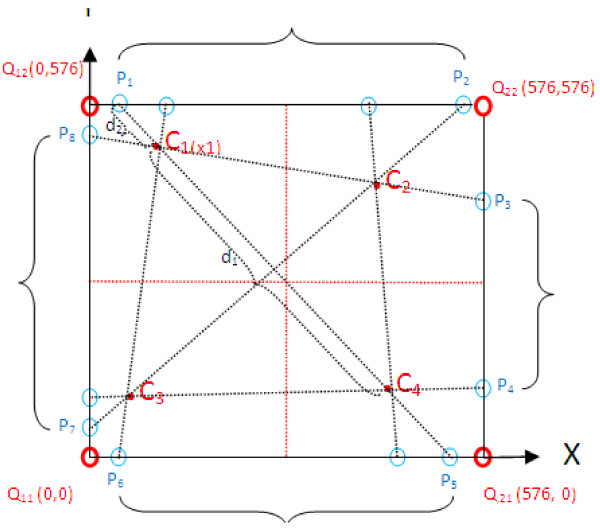
**Illustration of the bilinear interpolation approach.** The four red dots show the data click points and the red circled dots are the points at which we want to interpolate by using the calculated values (L values and xy coordinates values) of blue circled dots.

By taking into consideration the observed linear distribution, we opted for a bilinear interpolation scheme using four source points; this scheme allows to process images efficiently and produces good results. Let *C*1*, C*2*, C*3 and *C*4 be the four chosen points such that any three of them are not colinear as specified in Figure 13. For each pair of points, *Ci* and *Cj* , *i < j*, we first determine the intersection points of the line passing from these two points and the image boundaries; if both points are on the same border of the image, then the intersection points are assumed to be the points themselves. The mask values of the intersection points are then calculated by linear extrapolation of the L* values of the corresponding source points subtracted from the reference value. Once the mask values of intersection points are known, the mask value of any point on a border of the image can be found by calculating a set of projected values and taking their mean; each projected value is calculated by interpolating or extrapolating linearly the mask values of a pair of intersection points on that border. Finally, in order to find the mask value of any point (*x, y*) in the image, we apply linear interpolation in x and y directions separately using the mask values of the border points (i.e. interpolate the mask values of the two border points on column *x* and similarly on row *y*). Although each step is linear in the sampled values and in the position, the estimation as a whole is not linear but quadratic in the sample location.

**Figure 14:**
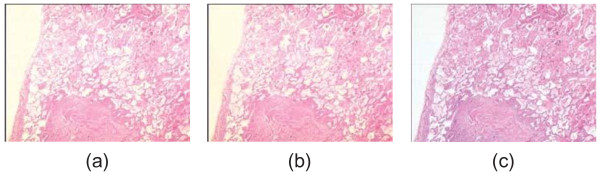
**Correction results. (a, b)** The resulting images after applying the proposed method to the sample images in Figure 10; **(c)** the resulting image when correction is applied in the RGB color model, red, green and blue separately (color separation in RGB) for the image in Figure 10(a) as it is applied for L* channel.

**Figure 15:**
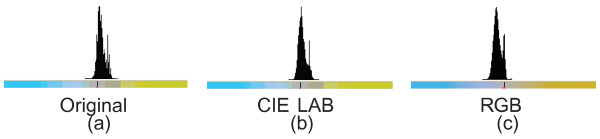
Illustration of the histograms for b* channel. (a) the histogram of the b* channel for the original image in Figure 10(a); **(b)** the histogram of the b* channel for the corrected image that is displayed in Figure 14(a) using Lab mode; **(c)** the histogram of the b* channel for the corrected image that is displayed in Figure 14(a) using RGB mode. This figure is displayed to reveal the variations before and after the correction in CIE LAB and RGB color models regarding the b* channel. Note that the center of the histogram for RGB model is moved to the left whereas the center of histogram for CIE LAB is not changed regarding the histogram of the original image.

Figure 14(a,b) shows the resulting images after applying the proposed method to the sample images in Figure 10 with illumination problems. One can observe that the uneven lighting (poor Koehler illumination) is removed from both images. Correcting them in RGB color model with similar algorithm applied on all channels separately causes a color shift (Figure 14c),^j^ demonstrating the advantage of using the CIE LAB color model. The corrected image (Figure 14c) in RGB mode has an appearance different from what the pathologists desire. This image doesn’t preserve the genuine color of H&E technique. The histograms of the b* channel of the sample image with uneven illumination (Figure 10a) before and after correction in CIE LAB and RGB color models are presented in Figure 15. The mean of the b* values for the first histogram is 133.32 and 130.65 for the second one whereas it is 118.75 for the third one that is processed in the RGB color mode. A color shift to the left is evident for the third histogram when it is compared to the b* values of the original image in Figure 15(a). The statistical significance analysis is performed on Matlab tool.^k^ Whether the variances of two normal distributions are equal is tested with the significance level of 0.05 using *F*-test with the two sided null hypothesis of $\;H_{0}:\sigma_{\text{bChannel}1}^{2}=\sigma_{\text{bChannel}2}^{2}$ where *bChannel1* indicates the b* values of the original image and *bChannel2* indicates the b* values of the corrected image. The high *p*-value, 0.611 (> 0.05), for comparing the b* values of images in Figures 10(a) and 14(a) means that there is no evidence against null hypothesis; that is to say, the observed outcome was likely enough that it is reasonable to assume that the null hypothesis is true so fail to reject the null hypothesis. On the other hand, there is a significant evidence that the images in 10(a) and 14(c) are not same $\;H_{0}:\sigma_{\text{bChannel}1}^{2}=\sigma_{\text{bChannel}3}^{2}$ (where *bChannel3* indicates the b* values of the corrected image using RGB mode) with a small *p*-value of 0.002 (< 0.05) in terms of the values distributed in b* channel. In brief, there is a nonsignificant difference between b* values of the original image and those of the one processed in the Lab color model. On the other hand there is a significant loss of essential information in 14(c) in terms of diagnosis by means of rejecting the null hypothesis $\;H_{0}:\sigma_{\text{bChannel}1}^{2}=\sigma_{\text{bChannel}3}^{2}$.

**Figure 16:**
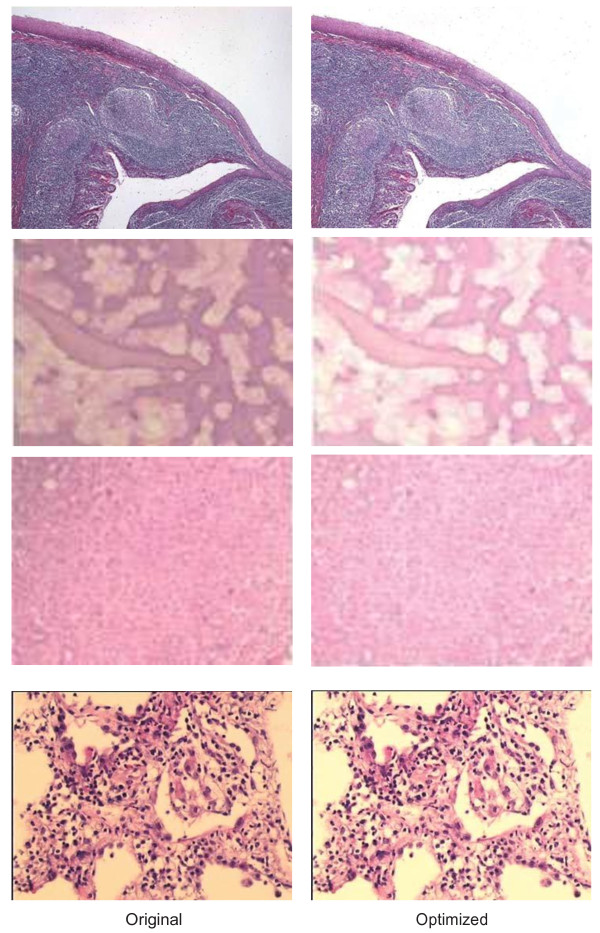
**Sample images before and after optimization in CIE LAB color model.** First column shows the original images whereas the second column shows the illumination correction of the images at first row. Chosen optimization factors mentioned in Figure 19 for each case are 5, 4, 3 and 2 respectively beginning from the upper case. Therefore, images are brighter with higher optimization factors.

Some resulting corrected images^l^ that are produced by the application are presented in Figure 16. Distinctive features of H&E technique in the images are made noticeably evident after the optimization of the illumination problem. No essential detail of nuclei and cytoplasm, in terms of diagnosis is lost thanks to the Lab color model and bilinear optimization method employed. Detail enhancement and sharpening can also be applied to Lab channels before converting the image back to the RGB color model to further improve the quality of the resulting image. One example of these is presented in the next section.

### Enhancing the delicate details in nuclei

We may explore some other possibilities for images of H&E stained microscopical materials by using Lab Color mode by adopting some basic image processing algorithms to other channels of Lab color mode, namely “a” and “b”. Unsharp mask is a classic sharpening technique, which was widely used by photographers before computer invented. The main idea is to blend image with blurred version. It emphasis edges and makes the image sharper. There are three parameters which are amount of sharpen, radius of blur, and threshold. The first parameter specifies how strong to sharpen image. The algorithm creates blurred version of the image and for each pixel calculates the difference between the original and the blurred image [16]. After that, it uses this difference to sharpen the image. The algorithm adds (difference × amount/100) to each pixel of the original image with amount specifying a percent of the difference to be added to the original image. The second parameter specifies radius of blur effect which creates defocused image. The higher value you enter, the wider will be edges. That’s why be careful when you adjust this parameter - a big radius will lead to unnatural effects (false halo around objects of the image). The third parameter specifies a threshold of the effect. If the difference between the pixels values of the original and the blurred images is less than the threshold, it is discarded. It allows to keep minor details unchanged and to apply sharpening only on noticeable details. For example, if you sharpen a photo which contains face, you would like to sharpen facial features (nose, lips, eyes, etc), but do not emphasize pimples, birthmark and other minor details. Zero is opted for the threshold to include all the minor details in this study. Here, an image processing algorithm is applied to both channels of L and b (nuclei) that is the prime important for pathologist to diagnose. In our study, some of the L* values are processed by referring only to b* values.

**Figure 17:**
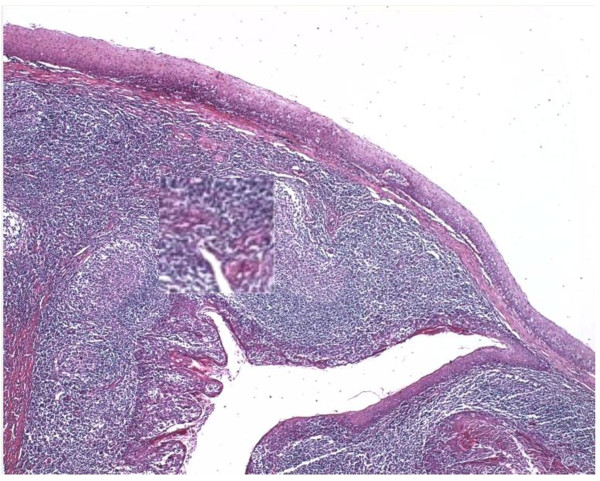
**First example for enhancement (optimized image).** The image is the product of the optimization method of the implementation. This image is used to perform the enhancement of nuclei method whose processed form is in Figure 20. A dedicated area on two images is magnified by ten times to perceive the effect of the method better.

**Figure 18:**
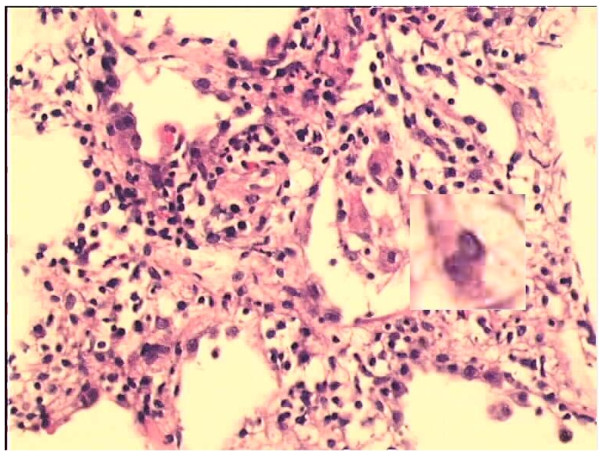
**Second example for enhancement (optimized image).** The image is the product of the optimization method of the implementation. This image is used to perform the enhancement of nuclei method whose processed form is in Figure 21. A dedicated area on two images is magnified by ten times to perceive the effect of the method better.

We did something similar to the unsharp mask mentioned above, however a little bit differently. Low pass filtering is employed to the channel of L by convolving optimized images in Figures 17 and 18 with a Gaussian kernel with a size of 3*3. The Gaussian function G(x, y) is defined in Eq.1:

(1) $$G\left(x,y\right)=\frac{1}{2\pi \sigma^{2}}\exp\left[-\left(\frac{x^{2}+y^{2}}{2\sigma^{2\;}}\right)\right]$$

**Figure 19:**
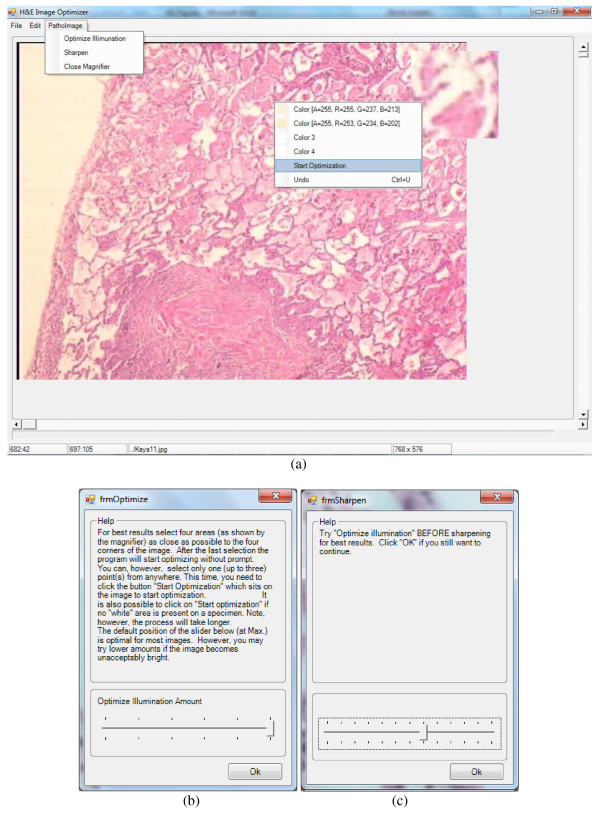
**Interface of the methodology. (a)** The area where the mouse sits is magnified 10 times to distinguish the white sections; the highlighted area is specified at the left bottom in terms of the pixel value where the mouse sits. The specified clicked points are displayed on the screen at the right click of the mouse to guide the user to select the next white point in the unclicked section of the quadrant. The last clicked pixel is chosen if the same section of the quadrant is selected more than once. **(b)** The small screen displayed after the selection of the function “Optimize Illumination”: the user should select an optimization factor up to 5; the larger the factor, the brighter the image is. The optimization process starts after the user clicks “Ok” button or “start optimization”. **(c)** The small screen displayed after the selection of the function “Sharpen”: the user should select a sharpening (enhancement) factor up to 12; the larger the factor, the sharper the blue sections are; the process starts after the user clicks “Ok” button. “undo” function is performed to reverse the image to its previous state to test using different optimization **(b)** and enhancement **(c)** factors.

**Figure 20:**
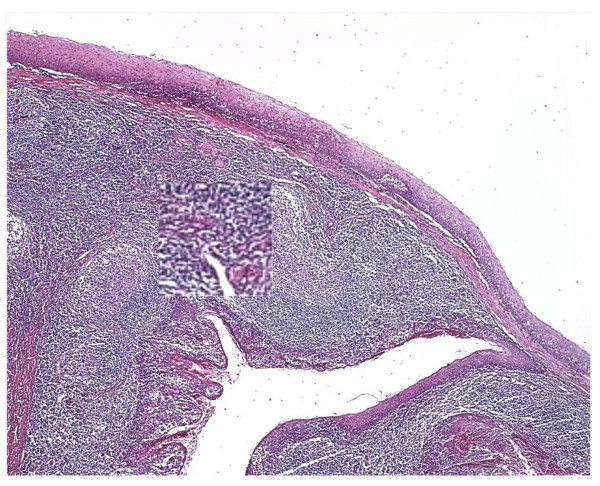
**First example for enhancement (enhanced image).** The image is the product of the enhancement of nuclei method performed for the image in Figure 17. Enhancement factor of 12 is used as mentioned in Figure 19. The magnified dedicated area should be examined to perceive the effect of the enhancement of nuclei method better. Note that only nuclei sections are enhanced.

**Figure 21:**
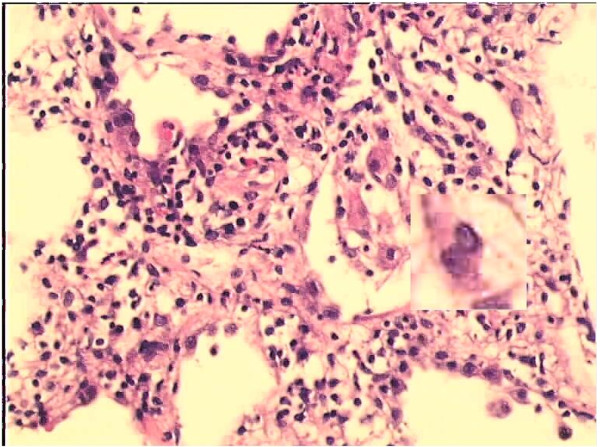
**Second example for enhancement (enhanced image).** The image is the product of the enhancement of nuclei method performed for the image in Figure 18. Enhancement factor of 7 is used as mentioned in Figure 19. The magnified dedicated area should be examined to perceive the effect of the enhancement of nuclei method better. Note that only nuclei sections are enhanced.

The effect of this function is to delimit the spatial frequencies in an image, resulting in loss of edge definition and averaging of intensity values; the larger the value of *σ*, the greater the smoothing effect [17]. We used a small default standard deviation of 3 after taking good results by applying different values to several images. The images obtained by the optimization method mentioned in the previous section (images in Figures 17 and 18) are convolved with the Gaussian kernel with a size or radius of 3*3 for L* channel. Radius is proportional to *σ*, the standard deviation of the Gaussian curve. Then, convolved L* channel is quantized between 0 and 100. The quantized L* channel of the image is extracted from the optimized *L* channel. Thus, extracted L* values of all the pixels in the images are obtained. We add these extracted values to the pixels in the optimized image with a multiplication value ranging from 1 to 12 which is chosen by the user to enhance the images more or less as depicted in Figure 19(c). Then we check if the b* channel values are greater or smaller than zero: it is the nuclei if the value of b* channel is smaller than zero. The pixel values of the desired image we want to observe are assigned with the values of the added multiplied extracted values to the optimized image if the b* channel is smaller than zero; else they are assigned with the same unchanged values of the optimized image. This second option of the methodology comes without bells&rings. It “sharpens” your image a little bit. Some of you may ask what is so special about this as the function is so widely available. Well, it actually tries to sharpen the “blue” objects (most likely nuclei) preferentially, b* channel. If the increased sharpness (which may cause a pixilation effect) looks irritating, the amount of sharpening should be reduced (Figure 19(c)). For the technically minded, the function does an “unsharp mask” on values of pixels in L* channel where these pixels overlap the bluish pixels (pixel values of b* *<* 0) using the Lab color model. In this respect, blue stained sections are enhanced by touching some of the pixels in L* channel. To see whether the image indeed got better, the enhanced images should be compared to the optimized ones; images in Figures 20 and 21 are sharpened/enhanced images which should be compared to the previous optimized ones, the images in Figures 17 and 18. In the comparison using the magnifier on a dedicated section on the images, it may be clearly noticed that the nuclei are also made crispier along with addressing the problem of illumination.

### Interface of the methodology

The proposed methodology has been implemented both in Java and in .NET.^m^ The interface of the application is depicted in Figure 19. The program allows the user to load an image acquired using the H&E staining technique, choose the source points and specify the reference L* values. Possible white areas can be seen easily in four quadrants of the images after they are highlighted by a magnification utility provided by the application since sometimes they are not evident by naked eye. Choosing points other than white areas may cause unexpected results. In order to facilitate the process of choosing suitable points, the program can highlight the probable white areas of the image (i.e. with L* values in CIE LAB model close to 100 and not smaller than 90^n^) and automatically determine the source points by dividing the image in four quadrants and picking in each quadrant the point among possible white points which is closest to the centroid of those points; if such a point does not exist in a quadrant, then the centroid of the points that are symmetric to the found points in other quadrants with respect to the XY axis is chosen as the source point and its mask value is set to the average of the mask values of the source points in other quadrants. “Correction of illumination” is implemented by the utility “optimize illumination” while “enhancing the details of nuclei” is implemented by the utility “sharpen” in the application. The course of illumination and enhancement can be adjusted using the factor selection screens in the application as displayed in Figure 19.

## Discussion

Microscopic digital imaging and telepathology have improved the practice of pathology in enabling examinations to be managed from remote locations. Telepathology allows pathologists to access second opinions in difficult cases as well as to participate in case discussions without traveling over long distances. Furthermore it allows hospitals without a pathologist to access expert opinions. However, microscopic digital images obtained from H&E technique suffer from uneven lighting. There are several common commercial methods that work in RGB mode to solve a widespread illumination problem generally occurred in photographs such as white balancing. These methods lead to a color shift in images which might not be very important for photographs. However, a color shift makes up an H&E image almost a different image on which it seems almost impossible to decide if the tissue has cancer cells. In addition to these commercial methods, several specific methods for remedying uneven illumination in microscopic images have been established. Some of the main drawbacks of these methods regarding H&E images are that it lacks in correcting the non-uniform illumination and it causes serious artifacts as mentioned in background section. These methods specific to microscopic images along with the commonly used commercial off-the-shelf methods are not successful to handle this kind of specific problem in H&E images. That’s why; these H&E images are left as they are as seen in medicine textbooks as well as in many publications. The problem needs to be solved specific to the features of the illumination problem and the features of the H&E images. This study by covering this specific problem area in pathology and histology presents a novel approach comprising both bilinear interpolation and Lab color mode to remedy the deficiencies of current methods. The effectiveness of the correction lies in quadratic estimation using XY plane as mentioned in the manuscript regarding Figure 13.

In the study, at first, the features of H&E images have been examined to reveal the patterns of illumination problems. In this respect, the distribution of illumination problem in a microscopic image is described. The perfect match between the H&E staining technique and Lab color mode is delineated and two methods employed in the channels of Lab separately as well as interrelated of channels are presented. The experimental results show that the illumination problem could be managed effectively and efficiently and delicate details can be enhanced not compromising precious data (H&E sections).

## Conclusions

In this study, an effective and efficient methodology for addressing the problem of uneven lighting in pathological images obtained using Hematoxylin & Eosin staining technique has been proposed. The method exploits the features of such images by first converting them into the CIE LAB color model, in which the lightness and the chromaticity of pixels can be modified independent of each other, and then employing a new approach along with bilinear interpolation to mask out the uneven lighting that spreads through an image. Furthermore, enhancement of H&E images is implemented by a novel approach together with unsharp mask. The empirical studies on a diverse set of sample images illustrated in the manuscript demonstrate that the proposed method is effective in improving the quality of H&E images; unlike other ad-hoc methods implemented in RGB color space, a color shift is not observed in the resulting images and crucial details are preserved. The established application provides pathologists with little or no image processing experience, an effective means of optimizing and enhancing H&E images for printing, image analysis, or telepathology.

### Future work

Some other basic image processing algorithms may be employed to other channels, a* and b*, of Lab color mode as well as L* channel as a future work. For instance, in terms of the chromaticity layers, a* and b*, the features of color problems of images could be analyzed. In this respect images should be captured from both non calibrated and calibrated microscopes and should be analyzed in order to recognize the patterns of color problems. New enhancement algorithms might be established to correct the problems in the chromaticity layers using acquired patterns of color problems.

## Endnotes

^a^Tissue samples may be obtained during surgery, after death or at a biopsy.

^b^Dyes are not pure substances; slight variations in manufacturing will have a profound effect on the final product; each manufacturer has its unique way to make a particular dye; constantly evolving health and safety regulations cause alterations in manufacturing protocols; recent geographic shifts in where dyes are made has had a major impact on the nature of dyes. Thus, sometimes it is not possible to see distinct blue or red color on images. Interested readers can reach more information about the H&E technique from the book whose editors are Kumar and Kiernan [2].

^c^The interested readers are referred to the articles written by Drent [3] and Fellers [18] for more information about the causes of both noise and poor Koehler illumination in digital microscopic images.

^d^Note that another name of auto-levels is white balancing.

^e^The plug-in can be downloaded from the web site, http://rsbweb.nih.gov/ij/plugins/inserm514/Documentation/A_posteriori_shading_correction_514_v3/A_posteriori_shading_correction_514_v3.html. Some sections of the image cannot be restored as explained at the website. Interested readers can reach some more examples from this website.

^f^The plug-in can be downloaded from the web site, http://www.optinav.com/Polynomial_Fit.htm.

^g^The plug-in can be downloaded from the web site, http://imagejdocu.tudor.lu/doku.php?id=plugin:filter:fit_polynomial:start.

^h^More examples can be reached from the web site, http://www.mecourse.com/landinig/software/cdeconv/cdeconv.html.

^i^Many images were obtained from a wide range of microscopes regarding the images we pulled out of publications and the images we obtained in our laboratory by employing several different set up options. All the microscopes from which our pathologists detected images are regularly well calibrated microscopes and very expensive ones in good quality.

^j^Correction is applied for all channels namely red, green and blue separately by color separation and then these channels are combined together to form an image.

^k^rgbImage1 = imread('specimen1.png');rgbImage2 = imread('corrected1.png');colorTransform = makecform('srgb2lab'); labImage1 = applycform(rgbImage1, colorTransform); labImage2 = applycform(rgbImage2,colorTransform);BChannel1 = labImage1(:, :, 3); BChannel2 = labImage2(:, :, 3);[h,p,CI,stats] = vartest2(double(BChannel1(:)),double(BChannel2(:)),0.05).

^l^Note that two of these images are the images depicted in Figure 1 on which several prominent methods are employed. A comparison of corrected illumination problem in these two images should be performed in terms of the proposed approach and the current prominent methods employed.

^m^The program can be downloaded from http://goo.gl/UGQR3.

^n^These values are not observed for the sections stained by H&E technique.

## Appendix

### Conversion between RGB and CIE LAB color models

In the study of color perception, one of the first mathematically defined color spaces was the CIE XYZ color space, created by the International Commission on Illumination (CIE) in 1931. CIE XYZ may be thought of as derived parameters from CIE RGB color space, the red, green, blue colors. CIE LAB color space is based directly on the CIE XYZ color space as an attempt to linearize the perceptibility of color differences. The non-linear relations for L*, a*, and b* are intended to mimic the logarithmic response of the eye. In order to convert an image from RGB color space to CIE LAB color space (or vice versa), the CIE XYZ color space is used as an intermediate color space at transformation phases from one color space into other [20].

The conversion formulas between CIE XYZ and RGB color spaces are as follows:

X=0.412453*R+0.357580*G+0.180423*B

Y=0.212671*R+0.715160*G+0.072169*B

Z=0.019334*R+0.119193*G+0.950227*B

R=3.240479*X−1.537150*Y−0.498535*Z

G=−0.969256*X+1.875992*Y+0.041556*Z

B=0.055648*X−0.204043*Y+1.057311*Z

The conversion formula from the CIE XYZ color space to the CIE LAB color space is defined as;

$$L*=116f\;\left(Y/Y_{n}\right)-16,$$

$$a*=500\left[f\;\left(X/X_{n}\right)\right]\;-f\;\left(Y/Y_{n}\right),$$

$$b*=200\left[f\;\left(Y/Y_{n}\right)\right]\;-f\;\left(Z/Z_{n}\right),$$

where *f* (*t*) = *t*^*1/3*^ for *t >* 0*.*008856, otherwise *f* (*t*) = 7*.*787 *t* + (16*/*116) and X_n,_ Y_n_ and Z_n_ denote the CIE XYZ tristimulus values of the reference white point. The division of the *f* (*t*) function into two domains is done to prevent an infinite slope at *t* = 0. *f* (*t*) is assumed to be linear below some *t* = *t*_0_, and is assumed to match the *t*^*1/3*^ part of the function at *t*0 in both value and slope.

$$t_{0}^{1/3}=t_{0}+b\left(\text{match}\;\text{in}\;\text{value}\right)\;\text{and}\;1/\left(3t_{0}^{2/3}\right)\;=a\left(\text{match}\;\text{in}\;\text{slope}\right)$$

In other words, the value of b was chosen to be 16*/*116. The above two equations can be solved for a and *t*0 to obtain

$$a=\frac{1}{3\delta }=7.787037\;\mathrm{and}\;t_{0}=\delta 3=0.008856$$

where *δ* = (6/29). The reverse transformation can then be calculated by applying the following rules:

1.

$$\mathrm{Define}\;f_{y}=\left(L*+16\right)/116,f_{x}=f_{y}+a*/500\;\mathrm{and}\;f_{z}=f_{x}-b*/200$$

2.

$$\mathrm{If}\;\mathrm{fy}>\delta \;\mathrm{then}\;Y=Y_{n}\;f_{y}^{3},\text{else}\;Y=\left(f_{y}-16/116\right)3\delta^{2}Y_{n}$$

3.

$$\mathrm{If}\;\mathrm{fx}>\delta \;\mathrm{then}\;X=X_{n}f_{x}^{3},\text{else}\;X=\left(\mathrm{fx}-16/116\right)3\delta^{2}X_{n}$$

4.

$$\mathrm{If}\;\mathrm{fz}>\delta \;\mathrm{then}\;Z=\mathrm{Zn}\;f_{z}^{3},\text{else}\;Z=\left(\mathrm{fz}-16/116\right)3\delta^{2}Z_{n}$$

## Competing interests

The author declares that he has no competing interests.
